# Spherocytosis-Related L1340P Mutation in Ankyrin Affects Its Interactions with Spectrin

**DOI:** 10.3390/life13010151

**Published:** 2023-01-04

**Authors:** Beata Machnicka, Aleksander Czogalla, Dżamila M. Bogusławska, Piotr Stasiak, Aleksander F. Sikorski

**Affiliations:** 1 Department of Biotechnology, Institute of Biological Sciences, University of Zielona Góra, 1 Prof. Z. Szafrana St., 65-516 Zielona Góra, Poland; 2 Department of Cytobiochemistry, Faculty of Biotechnology, University of Wrocław, 14a F. Joliot-Curie St., 50-383 Wrocław, Poland; 3 Department of Anatomy and Histology, Collegium Medicum, University of Zielona Góra, 28 Zyty St., 65-046 Zielona Gora, Poland; 4 Research and Development Centre, Regional Specialist Hospital, 73a Kamieńskiego St., 51-154 Wrocław, Poland

**Keywords:** erythrocyte membrane skeleton, ankyrin-R, spectrin, spectrin-binding domain, ankyrin–spectrin interaction, hereditary spherocytosis

## Abstract

Previously, we reported a new missense mutation in the *ANK1* gene that correlated with the hereditary spherocytosis phenotype. This mutation, resulting in L1340P substitution (HGMD CM149731), likely leads to the changes in the conformation of the ankyrin ZZUD domain important for ankyrin binding to spectrin. Here, we report the molecular and physiological effects of this mutation. First, we assessed the binding activity of human β-spectrin to the mutated ZZUDL1340P domain of ankyrin using two different experimental approaches—the study of association and dissociation responses of the spectrin–ankyrin binding domain and a sedimentation assay. In addition, we documented the changes in morphology caused by the overexpressed ankyrin ZZUD domain in human cell models. Our results prove the key role of the L1340 aa residue for the correct alignment of the ZZUD domain of ankyrin, which results in binding the latter with spectrin within the erythrocyte membrane. Replacing L1340 with a proline residue disrupts the spectrin-binding activity of ankyrin.

## 1. Introduction

The function of the erythrocyte membrane skeleton has been studied for years. Many interactions between membrane skeleton proteins as well as membrane skeleton proteins and integral membrane proteins have been discovered through the identification of naturally occurring mutants that are mostly associated with hemolytic anemias [1,2,3]. These mutants have been the subject of many studies to elucidate the regulatory mechanisms of the spectrin–ankyrin interaction, which has significantly increased our knowledge of the structure and function of the erythrocyte membrane skeleton [4,5,6,7,8,9,10].

Hereditary spherocytosis (HS), reported worldwide and characterized by the presence of spherical-shaped erythrocytes (spherocytes) on the peripheral blood smear, is the most common inherited anemia (1 in 2000 births) in individuals of Caucasian ancestry [11,12]. This erythrocyte membranopathy refers to a group of heterogeneous anemias that is most commonly associated with dominant inheritance, although non-dominant and recessive inheritance have also been described [13]. Many identified mutations have been found in genes encoding erythrocyte membrane proteins: ankyrin, anion exchanger 1 (AE1), β-spectrin, α-spectrin, or protein 4.2, defects of which lead to reduced deformability and loss of membrane surface area [14,15,16]. These in turn lead to reduced deformability due to the compromised integrity of the membrane skeleton [17]. The abnormal red blood cells (RBCs) are trapped and destroyed in the spleen, which is the main cause of hemolysis in HS [18]. Anemia, jaundice resulting from hemolysis and/or biliary obstruction, splenomegaly with reticulocytosis, and increased osmotic fragility of RBCs constitute the background of a family history of the disease [19].

Hereditary spherocytosis is, therefore, caused by mutations in one of the genes encoding RBC membrane proteins [11,20]. Overall, patients with dominantly inherited mutations are less affected than those with the least frequent (3–5% of cases) recessively inherited defects [1]. These cases are usually connected with a complete loss of protein or its function and greater destabilization of the erythrocyte membrane skeleton. With the rapid development and wide application of gene diagnostic technologies, the detection rate of HS cases is increasing [10,21]. Most of the mutations arise in exons, but some occur in introns, suggesting that intronic mutations also play a significant role in the pathogenesis of hereditary spherocytosis [22]. The use of whole-exome sequencing in recent times has made it possible to detect a particularly large number of new missense mutations, but most of them have not been described in terms of their functional consequences. Genetic screening in the Human Genome Mutation Database (HGMD) revealed several mutations in β-spectrin and ankyrin-R that lead to hereditary spherocytosis (http://www.hgmd.cf.ac.uk, accessed on 19 October 2022). To date, 57 different variants in the *SPTB* gene associated with the HS phenotype, including ten deletions, six insertions, twelve missense, fifteen nonsense, and eleven splicing mutations, were reported (HGMD; accessed on 6 October 2022). Interestingly, it is relatively easy to characterize the possible mutations in human HS of the gene *SPTB*, which are located within the ankyrin-binding domain: repeat 14 of β-spectrin—R 1756X [23] and repeat 15—A1884 V [24]. This domain and its direct interactions with the ZU5A subdomain of ankyrin-R are relatively well described [6,7,9]. Erythrocyte ankyrin contains an N-terminal membrane binding domain; a key spectrin-binding domain, which includes a highly conserved ZZU tandem; and a C-terminal regulatory domain, which contains the death domain [25,26,27,28]. Defects in ankyrin-R have been implicated in approximately half of all patients with hereditary spherocytosis [29,30]. In human hereditary spherocytosis, 93 different *ANK1* gene mutations, including 12 missense and 27 nonsense mutations, have been reported (HGMD; accessed on 6 October 2022), whereas 23 mutations were identified in the ZZUD tandem of ankyrin-R, including eight variants located in subdomain ZU5A, seven in ZU5B, three in UPA (R1252X, R1334X, and L1340P), and five in the death domain.

In our previous study, we reported a new missense mutation in the *ANK1* gene correlated with the HS phenotype in a three-generation Polish family with autosomal-dominant hereditary spherocytosis [31]. This mutation, resulting in L1340P (HGMD CM149731) substitution, probably leads to the changes in the ankyrin ZZUD domain interactions with spectrin. Leucine (L1340) is a conserved residue in human ankyrins (R, G, and B) [25] and is located in the UPA subdomain of ankyrin-R (NP_065209.2 ankyrin-1 isoform 1: L1340). Our previous modeling experiments showed that the missense mutation L1340P affects the secondary structure of the ZZUD tandem (NP_065209.2 ankyrin-1 isoform 1: 913-1487 https://www.uniprot.org/uniprotkb/P16157/entry, accessed on 6 October 2022) [31]. Moreover, the substitution allows for the rearrangement of several extra hydrogen bonds within the analyzed structure of the ZZUD tandem. As we have shown previously, two key phenylalanine residues, F913 and F916, are crucial for spectrin binding. The substitution of these residues led to a very large increase in the K_D_ value or abrogated binding completely [7]. The present study aims to identify the molecular and physiological effects of the mutation in the *ANK1* gene encoding the erythrocyte membrane protein ankyrin-R that leads to substitution with L1340P. Our data provide new insight into the regulatory mechanisms of the membrane skeletal proteins’ interaction pathways.

## 2. Materials and Methods

### 2.1. Plasmids and Bacterial Strains

The plasmid pEGFP-C1 and the cloning host *Escherichia coli* XL1-Blue strain were from Stratagene (USA), and the plasmid pRSETC, *E. coli* DH5α strain, and *E. coli* BL21(DE3)pLysE strain were from Invitrogen (USA).

### 2.2. Cloning, Expression, and Purification of His(6)GFP–AnkBD and GST-ZZUD Fragments and the L1340P Mutant

The ankyrin ZZUD supramodule was previously described as important for spectrin binding by ankyrin [31]. The 1746 bp length fragment of transcript variant 1 of the *ANK1* gene (NM_020476.3:2815–4560 bp) containing ankyrin ZZUD was synthesized at GenScript (International), employing *E. coli* codon optimization within the pUC57 plasmid and subsequently cloned into the pGEX-6P-1 high-expression vector using Bam-HI and Xho-I restriction enzymes and a standard ligation protocol. The GST-ZZUD construct was amplified in XL1-Blue *E. coli*, and positively screened plasmids were sequenced and transferred into the expression BL21(DE3)pLysS *E. coli* strain using a standard protocol. AnkBD of the spectrin 14–15 repeat was previously cloned into the pRSETC expression vector proceeded by a sequence encoding Green Fluorescent Protein obtained from the pEGFP-C1 [7].

Site-directed mutagenesis was performed with the QuickChange Site-Directed Mutagenesis Kit (Stratagene). The PCR products were stored in the *E. coli* strain XL1-Blue. The resulting plasmids bearing the mutated gene were sequenced to verify the mutations. The plasmid GST-ZZUDL1340P was then transferred into BL21(DE3)pLysS cells for overexpression of the proteins.

The overexpressed proteins GST-ZZUD, GST-ZZUDL1340P, and His(6)GFP–AnkBD were purified using Glutathione Sepharose 4B (GE Healthcare) or Talon (Clontech) resins accordingly, as described previously [6]. The concentration of the proteins was determined using a Cary 1E spectrophotometer employing extinction coefficient parameters determined for each protein [32]. The purity was also estimated from Coomassie stained SDS-PAGE on 10% gels (Figure S1).

### 2.3. CD Analysis

CD measurements were performed on a JASCO J-1500 CD spectrometer using a thermostat-controlled cell with a 0.2 cm path length from 20 to 70 °C in 5 °C increments within the range of 205–260 nm. The ellipticity values from the CD spectra were converted into molar residue ellipticity ([θ]M: in degrees cm^2^ dmol^−1^) values, as previous described [33]. Melting curves were generated with Boltzmann sigmoidal fit using GraphPad Prism software (GraphPad Software Inc., San Diego, CA, USA).

### 2.4. BLI Method

The Octet K2 2-channel System (ForteBio, Menlo Park, CA, USA) was used for the binding studies. The experiments were performed in black 96-well plates (Nunc F96 MicroWell Plates, (Thermo Fisher Scientific, Langenselbold, Germany). The total volume of each sample or buffer was 0.2 mL per well, and the shaking settings for every step were 1000 rpm. The test was performed at 30 °C. Before each assay, Ni^2+^-NTA biosensor tips (ForteBio, Menlo Park, CA, USA) were prewetted in 0.2 mL TRIS buffer (50 mM Tris–HCl, 150 mM NaCl, 2 mM DTT, pH 7.5) for at least 10 min. Subsequently, the equilibration step with TRIS buffer was performed for 60 s, then Ni^2+^-NTA biosensor tips were non-covalently loaded with His(6)GFP–AnkBD of spectrin for 600 s followed by an additional equilibration step (250 s) in TRIS buffer. The association step of His(6)GFP–AnkBD with GST-ZZUD and GST-ZZUDL1340P batches in a concentration of ZZUD (range 0–2.5 µM) was carried out for 300 s. Dissociation was the last step, and it lasted for 300 s. The response was measured as a shift in the interference pattern (in nm) and is proportional to the number of molecules bound to the surface of the biosensor. To analyze the association and dissociation responses, the curves were baseline corrected with the Octet Software (Version HT 11.1.0.25) and compared in terms of their amplitude for GST-ZZUD and GST-ZZUDL1340P.

### 2.5. Sedimentation Assay

The GST-tagged recombinant spectrin-binding domain of ankyrin (GST-ZZUD and GST-ZZUDL1340P) was adsorbed on a Glutathione Sepharose 4B resin via a GST tag. Equal volume aliquots of resin saturated with a GST-tagged protein were incubated with increasing concentrations (range 0–2 µM) of His(6)GFP–AnkBD of erythrocyte spectrin in the binding buffer (50 mM Tris–HCl, 150 mM NaCl, 2 mM DTT, pH 7.5) for 30 min at room temperature with gentle agitation. The resin was then spun down at 1000× *g* for 5 min, resuspended in elution buffer with 75 mM reduced GSH, and centrifuged again. The amount of bound His(6)GFP–AnkBD in the supernatant was determined using a Varian Cary Eclipse Spectrofluorimeter. The control experiments were performed using the Glutathione Sepharose 4B resin equilibrated in the test buffer and incubated with corresponding amounts of His(6)GFP–AnkBD. The bound values were obtained by subtracting the control values from those for His(6)GFP–AnkBD bound to the resin saturated with GST-ZZUD or GST-ZZUDL1340P. The binding parameters were identified using one site-specific binding fitting (GraphPad Software Inc., San Diego, CA, USA).

### 2.6. Resealed Erythrocyte Ghost Assay

Fresh blood samples were collected from healthy human volunteers (upon their informed, written consent) using anticoagulant (0.8% citric acid monohydrate, 2.2% trisodium citrate, 2.2% glucose). RBCs were washed three times with 10 mM Tris-HCl buffer (pH 7.4) containing 120 mM KCl. The intact cells were lysed in ice-cold lysis buffer (0.6 mM MgATP in 5 mM Tris-HCl, 5 mM KCl, 1 mM MgCl_2_, pH 7.4), centrifuged (15,000× *g*, 15 min, 4 °C), and washed in the same buffer until pale pink. The ghosts were resuspended in resealing buffer (150 mM KCl, 1.6 mM MgCl_2_, 1 mM DTT, 0.6 mM MgATP, pH 7.4) and incubated for 60 min at 37 °C in the presence or absence of recombinant ZZUD or its mutants (final concentration 1 mg/mL). After resealing, the suspension was centrifuged (10,000× *g*, 5 min, 4 °C), and the pellets and supernatants were collected for further analysis. The resealed ghosts were stained with lipophilic dye, Vybrant DiD (Molecular Probes), according to the manufacturer’s instructions, and observed on a fluorescent microscope (Zeiss AXIO), as described previously [34].

### 2.7. Cloning of ZZUD and Its L1340P Mutant Domain into mEGPF–C1 and mRFP-C1 Plasmids

Inserts of ZZUD and ZZUDL1340P were cloned out from a positive pGEX-6P-1-ZZUD or pGEX-6P-1-ZZUDL1340P with a PCR-based cloning procedure into a pEGFP-C1 (Addgene 54759) or mRFP-C1 (Addgene 54764) transfection vector coding for Green/Red Fluorescent Protein as a tag protein. The primers used for the PCR cloning are shown in Table 1. The positive clones were screened as described above using the primer combination (Table 2) EGFP–ZZUD, EGFP-ZZUDL1340P, and mRFP-C1-ZZUD; mRFP-C1-ZZUDL1340P plasmids were purified using a Plasmid Mini Kit (Eurex) and used for transfection.

### 2.8. HEL and K562 Cell Transfection

The HEL cell line was a gift from former Professor Marcin Majka from the Jagiellonian University School of Medicine and kept in our laboratory since 2010, as were K562 cells that were a kind gift from Professor D. Duś from the Institute of Immunology, Polish Academy of Sciences in Wrocław, Poland. The cell lines were regularly checked for Mycoplasma contamination. Before transfection, the HEL or K562 cells were grown directly on cover slips for 8 h in 6-well, 35 mm plates (Nunc Cell-Culture Treated Multidishes, Thermo Fisher Scientific) in RPMI 1640 and IMEM medium, respectively, with 10% FCS and antibiotics (100 I.U./mL streptomycin and 100 μg/mL penicillin), then transferred to medium without antibiotics. The cells were transfected with a Lipofectamine 2000 transfection reagent (Thermo Fisher) (μL) to DNA (μg) ratio of 3:1 mixture and cultured. Fluorescent microscopy assessed the changes in morphology resulting from the overexpression of WT or mutated ZZUD fragments after 48 and 72 h (Zeiss AXIO).

## 3. Results

### 3.1. Binding Studies of Ankyrin and Spectrin

**Figure 1:**
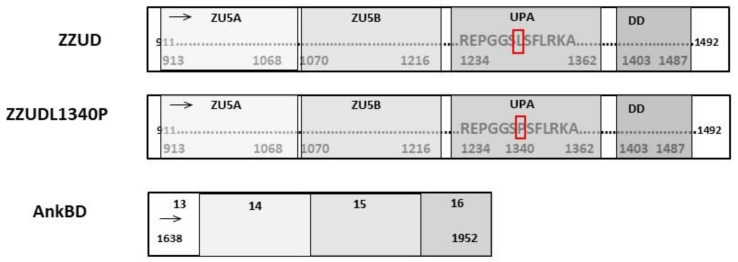
Schematic representation of the various ankyrin and spectrin fragments used in the assays concerning their length and domains.

The binding activity of β-spectrin was assigned to the ZZUD domain (ZU5-ZU5-UPA-DD) of ankyrin. ZZUD arranged into the following tandem (NP_065209.2 ankyrin-1 isoform 1: 913-1487 https://www.uniprot.org/uniprotkb/P16157/entry, accessed on 6 October 2022): two tandem ZU5 domains (ZU5A and ZU5B composed of 156 and 147 residues, respectively), subdomain UPA (129 residues), and DD (termed the death domain, which contains 85 residues) (Figure S3). Repeats 14–15 of β-spectrin are responsible for the interactions with the ZU5A subdomain of erythrocyte ankyrin [9]. The UPA domain could play a role in mediating the interactions between the ZU5B and DD domains [25,26]. To test the binding ability of the ankyrin ZZUD domain with spectrin, a fragment encompassing residues 911–1492 of ankyrin (NP_065209.2 ankyrin-1 isoform 1: 911-1492) was prepared. For the second construct, we introduced a point mutation within the ZZUD domain, which mimics a natural mutation described previously [31]. A graphical representation of the protein construct used is depicted in Figure 1.

Two independent approaches were used to measure and compare the binding activity of the spectrin to the ZZUD domain of ankyrin or its mutated version, ZZUDL1340P.

**Figure 2:**
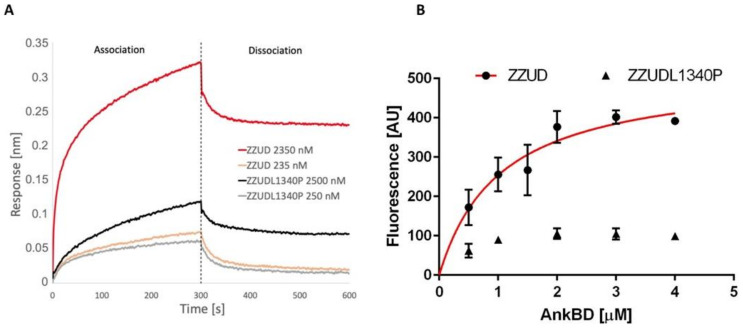
Quantification of binding between AnkBD and ZZUD or its mutated form. (**A**) Binding of ZZUD or ZZUDL1340P mutant to BLI sensor with AnkBD. (**B**) Saturation binding curve of ZZUD and AnkBD obtained via a sedimentation assay. N = 3; red curve was generated via one site-specific binding fitting.

In a BLI assay, we measured the association and dissociation responses of the ZZUD domain of ankyrin or its mutant to AnkBD of spectrin immobilized to the sensors using the two-channel Octet K2 system (Figure 2A). We observed a significant decrease (approx. threefold) in the binding response in the case of the ankyrin fragment bearing the L1340P mutation compared to the WT counterpart. This strongly suggests that ankyrin–spectrin interactions may be corrupted due to the L1340P mutation.

To obtain more quantitative data on the influence of the L1340P mutation on spectrin–ankyrin interactions in parallel to BLI, we employed a sedimentation assay (Figure 2B) using GFP-labelled AnkBD of spectrin and the GST-ZZUD domain of ankyrin. Having saturation binding curves, we managed to calculate the equilibrium dissociation constant. The value of the equilibrium dissociation constant K_D_ = 1.039 ± 0.27 µM indicates that AnkBD of spectrin and the ZZUD domain of ankyrin complexes of high affinity are formed, and the value is similar to earlier values reported by others [4,25,35] or by us [7]. However, for the mutated form of the ankyrin-derived fragment (ZZUDL1340P), it was not possible to calculate the K_D_ due to the very low fluorescence values obtained (Bmax approx. 5-times lower than in the case of the wild-type domain). These results showed that the presence of the L1340P mutation in the ZZUD domain significantly disrupts the ability to form a high-affinity complex. Moreover, the results obtained from the ZZUDL1340P fragment further confirm the importance of the leucine residue in the proper binding of spectrin with ankyrin, as we suggested previously [31].

The circular dichroism (CD) analysis confirmed that the loss of binding was not caused by the instability of the mutant. The actual T_m_ of the mutant was higher than that for the WT ZZUD domain (Figure S2B). Both protein domains are characterized by a high level of stability and undergo similar folding. AnkBD of spectrin was also confirmed to be properly folded (Figure S2). The recorded CD spectra might suggest that the L1340P mutation results in some very minor changes in the secondary structure, although further structural studies would be needed to confirm this suggestion (Figure S2). Slight changes in one of the β-sheets were also visible in the model, which we previously generated [31]. The modelling experiments performed there showed that the substitution L1340P affects structural changes in the beta strand, which is longer, and the orientation of the F913 residue is changed. Such a change would not be visible in the CD spectra.

### 3.2. Effect of Mutations on the Morphology of Resealed Erythrocyte Ghosts

**Figure 3:**
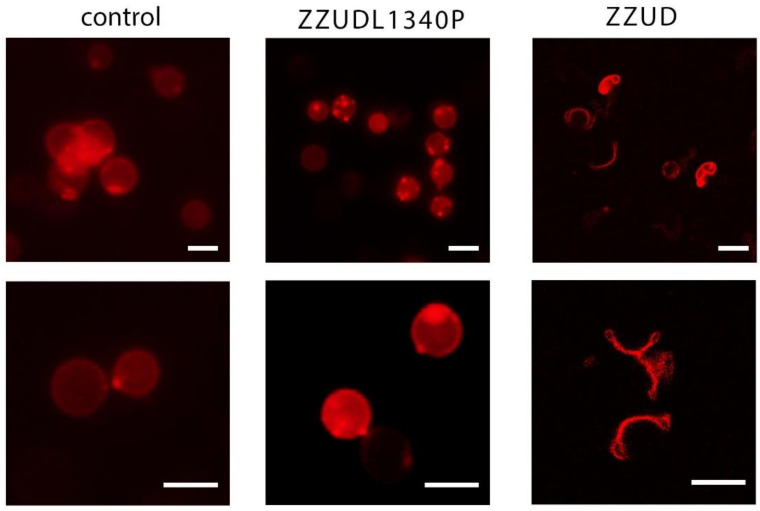
Effect of the ZZUD domain and its mutant form on the morphology of the resealed erythrocyte ghosts. The ghosts of the erythrocytes loaded with the non-mutated ZZUD domain were characteristically deformed (right panel), while those loaded with the purified mutated ZZUD domain remained spherical (central panel). In the left panel was shown normal morphology of the erythrocyte ghosts. Scale bar 5 µm.

The effect of encapsulation of the WT or the mutated ZZUD domain of ankyrin in the erythrocyte ghost on their morphology was investigated. For this purpose, red blood cells were prepared, loaded with the purified wild-type ZZUD domain and its L1340P mutant, and then the resealed ghosts were stained with DiD dye. It was observed that the ghosts of the erythrocytes loaded with the non-mutated ZZUD domain were characteristically deformed, while those loaded with the purified mutated ZZUDL1340P domain did not change their shape (Figure 3). These results indicate the lack of the ability of ZZUDL1340P to compete for binding erythrocyte ghost spectrin with native ankyrin residing within the membrane skeleton responsible for shape maintenance. The lack of changes in the erythrocyte ghost morphology in the presence of the mutant form suggests the importance of the mutant L1340P residue in maintaining the mechanical properties of the membrane and the shape of the erythrocytes.

### 3.3. Effects of Mutations on the Shape of Transiently Transfected HEL and K562 Cells

To assess the effect of overexpression of the spectrin-binding domain of ankyrin ZZUD on living cells in vitro, HEL and K562 cells were transiently transfected with vectors carrying inserts encoding protein fragments fused with GFP or RFP as described in the Materials and Methods section. The changes in morphology resulting from the overexpression of ZZUD or mutated ZZUDL1340P fragments of ankyrin were observed with fluorescent microscopy.

**Figure 4:**
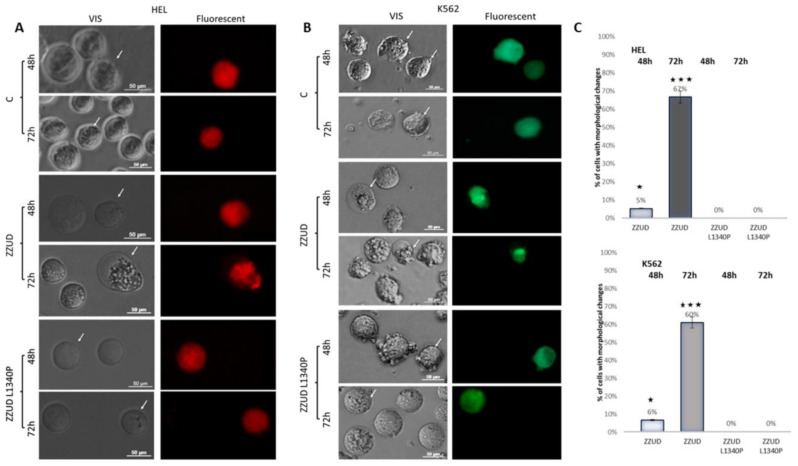
HEL (panel **A**) and K562 (panel **B**) cells were transfected with EGFP/RFP ZZUD and EGFP/RFP ZZUDL1340P plasmids and imaged 48 and 72 h after transfection. The cells continue to have a circular outline, but the ankyrin-derived fragments accumulate in a certain region of the cell, leading to markedly changed morphology; such appearance is not present in the wild-type/control cells or those transfected with the ZZUDL1340P bearing vector. This increased number of cells with altered morphology was highly statistically significant as shown in panel (**C**) (★, *p* < 0.05; ★★★, *p* < 0.005).

For comparison, the constructs carrying the spectrin-binding domain or its L1340P mutant were overexpressed in the erythroblast cell line HEL. The results are presented in Figure 4A. HEL cells transfected with the vector encoding fluorescent protein only were used as a control. The first effect of overexpression was observed 48 h after transfection. RFP and RFP-ZZUDL1340P overexpression did not change the morphology of the cells. In contrast, the overexpression of erythroid ankyrin’s ZZUD (wild type) caused granulation along the membrane surface. The effect was more visible 3 days after transfection (67% of cells had dramatically changed morphology). Four days after transfection, almost all the wild-type ZZUD transfected cells were dead, in contrast with the cells overexpressing the mutated fragment of ankyrin. The cells transfected with the control plasmid and a plasmid carrying an insert encoding the mutated ZZUDL1340P domain remained alive throughout the time of the experiment without any changes in cell shape.

Similar observations were made using the myeloid cell line K562 (Figure 4B). The overexpression of the ZZUD domain caused changes in cell morphology 48 and 72 h after transfection in 6% and 60%, respectively. The observed cells had an increasing aggregation pattern.

These results indicate that the wild-type ZZUD domain, when transiently overexpressed in living cells, induced dramatic changes in the spectrin-based membrane skeleton, leading to substantial changes in cell morphology, while no effect was observed for the ZZUD L1340P mutant (see also Figure 4C).

## 4. Discussion

In hereditary spherocytosis (HS), the inappropriate binding of spectrin to ankyrin is highly correlated with the severity of the disease [15,25]. The region responsible for proper spectrin binding is the ZU5A subdomain of erythrocyte ankyrin, which interacts with repeats 14–15 of β-spectrin [4,5].

The structural studies indicate that the second ZU5B domain is involved in the formation of an auto-regulating supramodule of ankyrin, ZZUD [26]. Based on the analysis of eight ankyrin-B syndrome-causing mutations (including two located in the UPA domain), Wang et al. suggested that the ankyrin-B ZZUD tandem is likely to bind to other proteins, thereby regulating its N-terminal ankyrin repeat-mediated membrane binding. Another missense mutation in ankyrin-R, W1185R [25], which is located in the ZU5B domain and not directly involved in spectrin binding, can cause hereditary spherocytosis. This further indicates that this ZU5 domain plays an important functional role within ankyrins.

The main factor in the correct binding of ankyrin and spectrin is the presence of complementary charged surfaces on these proteins that match each other [7]. Pathological lesions in HS result mainly from the loss of spectrin and/or ankyrin that is normally responsible for anchoring the membrane skeleton in the lipid bilayer. The current knowledge is constantly updated with new mutations resulting in the HS phenotype [10,36,37,38,39].

In our previous study, we described a new missense mutation resulting in L1340P (HGMD CM149731) substitution in the *ANK1* gene that correlated with the HS phenotype [31]. The aim of this project was to perform in vitro experimental analyses using purified ankyrin and spectrin protein domains previously described as being involved in interactions with each other and to determine the impact of the L1340P mutation found in ankyrin on the interaction between these proteins. For the purposes of the study, a stable construct of ZZUD fragments of ankyrin with a mutation, which mimics a natural mutation L1340P, was prepared. The applied strategy allowed us to purify bacterially expressed and properly folded proteins. The CD spectra suggest that both fragments correspond to the native protein. The binding activity of human β-spectrin with the mutated ZZUDL1340P domain of ankyrin was assessed using two different experimental approaches—a sedimentation assay and a study of association and dissociation responses with respect to the spectrin–ankyrin binding domain via biolayer interferometry. The obtained K_D_ values of the ZZUD domain–AnkBD of spectrin complexes roughly corresponded to the values obtained previously for interaction between spectrin and ankyrin by different teams [6]. The binding affinity of spectrin to the mutated ZZUDL1340P domain decreased dramatically when compared to the control wild-type ZZUD domain.

In our next experiment, the lack of the ability of the mutated fragment of ZZUD to compete for binding with erythrocyte ghost spectrin was demonstrated. The absence of changes in the erythrocyte ghost morphology in the presence of the mutant form suggests that the mutant L1340P does not compete for spectrin with native ankyrin. Thus, the amino acid residue at the 1340 position is critical in maintaining the mechanical properties of the membrane and the shape of the erythrocytes. Moreover, in cellular models, we demonstrated changes in the morphology of the cells overexpressing the ZZUD domain of ankyrin. In the transfected erythroblast cell line, HEL, and the myeloid cell line, K562, the expression of GFP/RFP-ZZUD proteins causes large extensive granulation along the membrane surface of both cell types. Probably, the spectrin-binding fragment of ankyrin is competitive for endogenous spectrin in ankyrin binding activity. It should be noted that with time, the number of cells overexpressing the ZZUD domain dramatically decreased. This was probably caused by the enhanced mortality of these cells due to the presence of more ankyrin fragments, which destabilized the membrane. We observed a similar phenotype previously in HeLa cells overexpressing the binding domain of spectrin [6].

In summary, our in vitro and in-cell results prove the key role of L1340 in the UPA domain for the proper function of the ZZUD domain, resulting in the ability to form a bond between spectrin and ankyrin in the erythrocyte membrane. Replacing L1340 with a proline residue disrupts the activity of ankyrin to bind spectrin, which is correlated with the HS phenotype of patients with autosomal-dominant hereditary spherocytosis. Thus, the hypothesis concerning the effect of a mutation was confirmed via experiments filling the gap between the genetic data and the physiological effect observed at the level of the whole organism.

## Figures and Tables

**Table 1:** Primers used for PCR cloning.

Primer	Sequence
Sense Strand	ZZUD Xhol 5′-GACCTCGAGCGTTCCTGGTTTCTTTTATGG-3′
Antisense strand	ZZUD HindIII 5′-CGTCAAGCTTCAACCACTACCTTCCAGC-3′

**Table 2:** Primers used for sequencing.

	EGFP-ZZUD/ZZUDL1340P	mRFP1-ZZUD/ZZUDL1340P
Sense strand primers	EGFP-C 5′-CATGGTCCTGCTGGAGTTCGTG-3′	DsRed1-C 5′-AGCTGGACATCACCTCCCACAACG-3′
	SV40p-A-Rs 5′-CCACGAAACTGGTGTATGC-3′	RFP-2s 5′-GTCTGCTGTGCTCTGTGATTGG-3′
Antisense strand primers	EGFP-Ca 5′-CTGCCGTCAGTTCTTTATACAGC-3′	RFP-1a 5′-GACAATCGGACAGCCAGAAACG-3′
	SV40p-A-R 5′-GAAATTTGTGATGCTATTGC-3′	RFP-2a 5′-AACCTCTACAAATGTGGTATGGC-3′

## Data Availability

All sequences have been reported in Supplementary Materials. Further information on experimental detail are available from authors on reasonable request.

## Supplementary Materials

The following supporting information can be downloaded at: https://www.mdpi.com/article/10.3390/life13010151/s1, Figure S1: Representative SDS-PAGE of purified His(6)GFP-AnkBD (**A**) and ZZUD-GST (**B**) and ZZUDL1340P-GST (**C**). Figure S2. Representative circular dichroism spectra (**A**) measured at 20 °C and melting curves (**B**) of His(6)GFP-AnkBD (blue), ZZUD-GST (red) and ZZUD L1340-GST (gray). Figure S3. The sequence of ZZUD supramodule according UNIPROT database https://www.uniprot.org/uniprotkb/P16157/entry, accessed on 19 October 2022.

## References

[1] Narla J., Mohandas N. (2017). Red cell membrane disorders. Int. J. Lab. Hem..

[2] Pugi J., Drury L., Langer J., Butchart S., Fantauzzi M., Baker J., Blanchette V., Kirby-Allen M., Carcao M. (2016). Genotype/Phenotype Correlations in 103 Children from 87 Families with Hereditary Spherocytosis. Blood.

[3] Mohandas N. (2018). Inherited hemolytic anemia: A possessive beginner’s guide. Hematology.

[4] Ipsaro J., Huang L., Mondragón A. (2009). Structures of the spectrin-ankyrin interaction binding domains. Blood.

[5] Ipsaro J., Mondragón A. (2010). Structural basis for spectrin recognition by ankyrin. Blood.

[6] Kolondra A., Grzybek M., Chorzalska A., Sikorski A. (2008). The 22.5kDa spectrin-binding domain of ankyrinR binds spectrin with high affinity and changes the spectrin distribution in cells in vivo. Protein Expr. Purif..

[7] Kolondra A., Lenoir M., Wolny M., Czogalla A., Overduin M., Sikorski A., Grzybek M. (2010). The role of hydrophobic interactions in ankyrin–spectrin complex formation. Biochim. Biophys. Acta (BBA)-Biomembr..

[8] La-Borde P., Stabach P., Simonović I., Morrow J., Simonović M. (2010). Ankyrin recognizes both surface character and shape of the 14–15 di-repeat of β-spectrin. Biochem. Biophys. Res. Commun..

[9] Czogalla A., Sikorski A. (2010). Do we already know how spectrin attracts ankyrin?. Cell. Mol. Life Sci..

[10] Bogusławska D., Skulski M., Machnicka B., Potoczek S., Kraszewski S., Kuliczkowski K., Sikorski A. (2021). Identification of a Novel Mutation of β-Spectrin in Hereditary Spherocytosis Using Whole Exome Sequencing. Int. J. Mol. Sci..

[11] Delaunay J. (2007). The molecular basis of hereditary red cell membrane disorders. Blood Rev..

[12] Da Costa L., Galimand J., Fenneteau O., Mohandas N. (2013). Hereditary spherocytosis, elliptocytosis, and other red cell membrane disorders. Blood Rev..

[13] Agre P., Asimos A., Casella J., McMillan C. (1986). Inheritance Pattern and Clinical Response to Splenectomy as a Reflection of Erythrocyte Spectrin Deficiency in Hereditary Spherocytosis. N. Engl. J. Med..

[14] Bogusławska D., Heger E., Baldy-Chudzik K., Zagulski M., Maciejewska M., Likwiarz A., Sikorski A. (2006). (AC)n microsatellite polymorphism and 14-nucleotide deletion in exon 42 ankyrin-1 gene in several families with hereditary spherocytosis in a population of South-Western Poland. Ann. Hematol..

[15] Eber S., Lux S. (2004). Hereditary spherocytosis—Defects in proteins that connect the membrane skeleton to the lipid bilayer. Semin. Hematol..

[16] Paździor G., Langner M., Chmura A., Bogusławska D., Heger E., Chorzalska A., Sikorski A. (2003). The kinetics of haemolysis of spherocytic erythrocytes. Cell Mol. Biol. Lett..

[17] Demiralp D., Peker S., Turgut B., Akar N. (2012). Comprehensive identification of erythrocyte membrane protein deficiency by 2D gel electrophoresis based proteomic analysis in hereditary elliptocytosis and spherocytosis. Prot. Clin. Appl..

[18] Reliene R., Mariani M., Zanella A., Reinhart W., Ribeiro M., del Giudice E., Perrotta S., Iolascon A., Eber S., Lutz H. (2002). Splenectomy prolongs in vivo survival of erythrocytes differently in spectrin/ankyrin- and band 3-deficient hereditary spherocytosis. Blood.

[19] King M.-J., Zanella A. (2013). Hereditary red cell membrane disorders and laboratory diagnostic testing. Int. J. Lab. Hematol..

[20] Tse W., Lux S. (1999). Red blood cell membrane disorders. Br. J. Haematol..

[21] Li H., Papageorgiou D., Chang H.-Y., Lu L., Yang J., Deng Y. (2018). Synergistic Integration of Laboratory and Numerical Approaches in Studies of the Biomechanics of Diseased Red Blood Cells. Biosensors.

[22] He B.-J., Liao L., Deng Z.-F., Tao Y.-F., Xu Y.-C., Lin F.-Q. (2018). Molecular Genetic Mechanisms of Hereditary Spherocytosis: Current Perspectives. Acta Haematol..

[23] Maciag M., Płochocka D., Adamowicz-Salach A., Burzyńska B. (2009). Novel beta-spectrin mutations in hereditary spherocytosis associated with decreased levels of mRNA. Br. J. Haematol..

[24] Gallagher P., Forget B. (1998). Hematologically Important Mutations: Spectrin and Ankyrin Variants in Hereditary Spherocytosis. Blood Cells Mol. Dis..

[25] Wang C., Yu C., Ye F., Wei Z., Zhang M. (2012). Structure of the ZU5-ZU5-UPA-DD tandem of ankyrin-B reveals interaction surfaces necessary for ankyrin function. Proc. Natl. Acad. Sci. USA.

[26] Yasunaga M., Ipsaro J., Mondragón A. (2012). Structurally Similar but Functionally Diverse ZU5 Domains in Human Erythrocyte Ankyrin. J. Mol. Biol..

[27] Xia X., Liu S., Zhou Z. (2022). Structure, dynamics and assembly of the ankyrin complex on human red blood cell membrane. Nat. Struct. Mol. Biol..

[28] Vallese F., Kim K., Yen L., Johnston J., Noble A., Calì T., Clarke O. (2022). Architecture of the human erythrocyte ankyrin-1 complex. Nat. Struct. Mol. Biol..

[29] Gallagher P. (2005). Hematologically important mutations: Ankyrin variants in hereditary spherocytosis. Blood Cells Mol. Dis..

[30] Agarwal A. (2019). Ankyrin Mutations in Hereditary Spherocytosis. Acta Haematol..

[31] Bogusławska D., Heger E., Listowski M., Wasiński D., Kuliczkowski K., Machnicka B., Sikorski A. (2014). A novel L1340P mutation in the ANK1 gene is associated with hereditary spherocytosis?. Br. J. Haematol..

[32] Gasteiger E., Hoogland C., Gattiker A., Duvaud S., Wilkins M., Appel R., Bairoch A., Walker J. (2005). Protein Identification and Analysis Tools on the ExPASy Server. The Proteomics Protocols Handbook.

[33] Bilkova E., Pleskot R., Rissanen S., Sun S., Czogalla A., Cwiklik L., Róg T., Vattulainen I., Cremer P., Jungwirth P. (2017). Calcium Directly Regulates Phosphatidylinositol 4,5-Bisphosphate Headgroup Conformation and Recognition. J. Am. Chem. Soc..

[34] Chorzalska A., Łach A., Borowik T., Wolny M., Hryniewicz-Jankowska A., Kolondra A., Langner M., Sikorski A. (2010). The effect of the lipid-binding site of the ankyrin-binding domain of erythroid β-spectrin on the properties of natural membranes and skeletal structures. Cell. Mol. Biol. Lett..

[35] Ipsaro J., Huang L., Gutierrez L., MacDonald R. (2008). Molecular Epitopes of the Ankyrin−Spectrin Interaction. Biochemistry.

[36] Chai S., Jiao R., Sun X., Fu P., Zhao Q., Sang M. (2020). Novel nonsense mutation p. *Gln*264Ter in the ANK1 confirms causative role for hereditary spherocytosis: A case report. BMC Med. Genet..

[37] Hao L., Li S., Ma D., Chen S., Zhang B., Xiao D., Zhang J., Jiang N., Jiang S., Ma J. (2019). Two novel *ANK1* loss-of-function mutations in Chinese families with hereditary spherocytosis. J. Cell Mol. Med..

[38] Russo R., Andolfo I., Manna F., Gambale A., Marra R., Rosato B., Caforio P., Pinto V., Pignataro P., Radhakrishnan K. (2018). Multi-gene panel testing improves diagnosis and management of patients with hereditary anemias. Am. J. Hematol..

[39] Bogusławska D., Heger E., Machnicka B., Skulski M., Kuliczkowski K., Sikorski A. (2017). A new frameshift mutation of the β-spectrin gene associated with hereditary spherocytosis. Ann. Hematol..

