# Supplementary material for: Spherocytosis-Related L1340P Mutation in Ankyrin Affects Its Interactions with Spectrin
Source: Life (Basel). 2023 Jan 4;13(1):151. doi: 10.3390/life13010151 (PMC9864249; doi:10.3390/life13010151)
Supplement: Supplementary file 1 [file life-13-00151-s001.zip › life-1986481-supplementary.pdf]

Spherocytosis-Related L1340P Mutation in Ankyrin Affects Its Interactions with Spectrin

Beata Machnicka, Aleksander Czogalla, Dżamila M. Bogusławska, Piotr Stasiak and Aleksander F. Sikorski

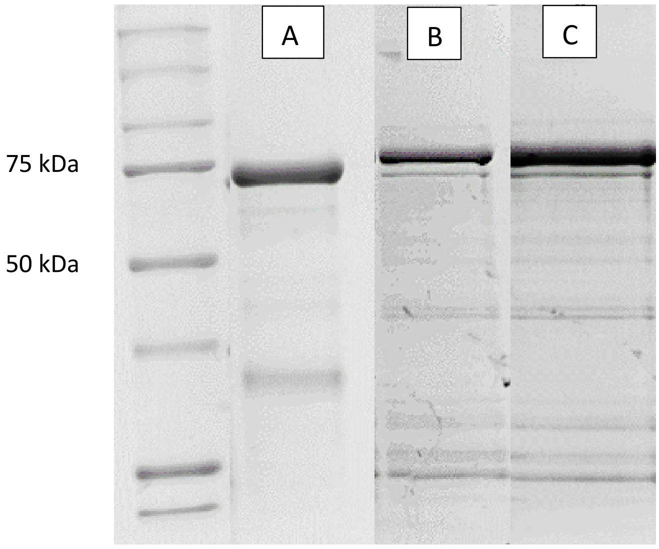

Figure S1. Representative SDS-PAGE of purified His(6)GFP-AnkBD (A) and ZZUD-GST (B) and ZZUDL1340P-GST (C). Samples were run in 10% gel and stained with Coomassie Blue. Standard was Precision Plus Unstained Protein Standard (Bio-Rad).

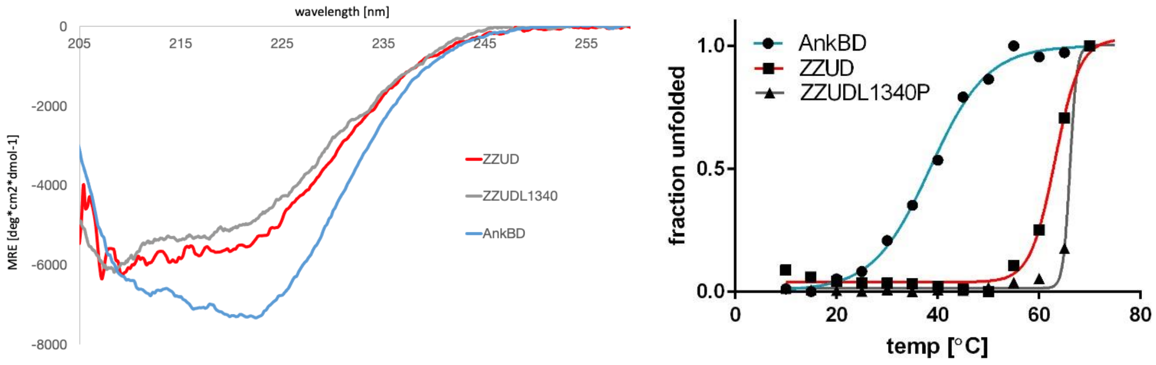

(**A**) (**B**)

Figure S2. Representative circular dichroism spectra (**A**) measured at 20 °C and melting curves (**B**) of His(6)GFP-AnkBD (blue), ZZUD-GST (red) and ZZUD L1340-GST (gray).

ZU5 1
>sp|P16157|ANK1_HUMAN|913-1068 OS=Homo sapiens OX=9606 GN=ANK1 PE=1 SV=3
FLVSFMVDAR GGSMRGSRHN GLRVVIPPRT CAAPTRITCR LVKPQKLSTP PPLAEEEGLA
SRIIALGPTG AQFLSPVIVE IPHFASHGRG DRELVVLRSE NGSVWKEHRS RYGESYLDQI
LNGMDEELGS LEELEKKRVC RIITTDFPLY FVIMSR
ZU5 2
>sp|P16157|ANK1_HUMAN|1070-1216 OS=Homo sapiens OX=9606 GN=ANK1 PE=1 SV=3
CQDYDTIGPE GGSLKSKLVP LVQATFPENA VTKRVKLALQ AQPVPDELVT KLLGNQATFS
PIVTVEPRRR KFHRPIGLRI PLPPSWTDNP RDSGEGDTTS LRLLCSVIGG TDQAQWEDIT
GTTKLVYANE CANFTTNVSA RFWLSDC
UPA
>sp|P16157|ANK1_HUMAN|1234-1362 OS=Homo sapiens OX=9606 GN=ANK1 PE=1 SV=3
TAVPYMAKFV IFAKMNDPRE GRLRCYCMTD DKVDKTLEQH ENFVEVARSR DIEVLEGMSL
FAELSGNLVP VKKAAQQRSF HFQSFRENRL AMPVKVRDSS REPGGSLSFL RKAMKYEDTQ
HILCHLNIT
Death
>sp|P16157|ANK1_HUMAN|1403-1487 OS=Homo sapiens OX=9606 GN=ANK1 PE=1 SV=3
AEMKMAVISE HLGLSWAELA RELQFSVEDI NRIRVENPNS LLEQSVALLN LWVIREGQNA
NMENLYTALQ SIDRGEIVNM LEGSG

Figure S3. The sequence of ZZUD supramodule according UNIPROT database https://www.uniprot.org/uniprotkb/P16157/entry.
